# A Note on the Nature of Neurasthenia

**Published:** 1910-09

**Authors:** Tom A. Williams

**Affiliations:** Washington, D. C.; M. Cor. Soc. de Neurol de Paris, etc., Neurologist to Epiphany Free Dispensary


### A NOTE ON THE NATURE OF NEURASTHENIA.

BY DR. TOM A. WILLIAMS, WASHINGTON, D. C.,

*M. Cor. Soc. de Neurol de Paris, etc., Neurologist to Epiphany Free Dispensary.*

A longitudinal view-point rather than a horizontal one should be taken in studying this disease. That method, in psychiatry, has been most fruitful, enabling us to separate from a vast unknown mass, the convenient syndromes known as dementia precox and manic-depressive insantty.

In what is often called neurasthenia, close analysis generally reveals some other condition, more usually involutional melancholia, psychasthenia or hysteria.

The first of these, and neurasthenia, may each take a long time, or may recover rapidly. The difference is sometimes one of intensity, rather than of kind. The fixity of the false ideas is a somewhat arbitrary distinction, as they are not always in reality fixed, the patient usually being susceptible to argument, for the moment at least, even when inveterately melancholic.

In psychasthenia, there is no morbid physical fatiguability as a rule, even in patients whose will-power may be annihilated. The obsessions of fatigue neuroses are only evanescent too, and are not fixed into the patient's constitution for years, as are sometimes those of the psychasthenic, the constitutional psychasthenic in particular. In severe neurasthenia, psychastheniform symptoms may show themselves for a time; but they are secondary to the bodily disturbances and disappear with these. (See Tri-State Med. Ass. 1909 Transactions. Author's article on *Dif. Treatment for Conditions often Mistaken for Neurasthenia*; also Archives of Diagnosis, Jan., 1909, Charlotte Med. Jour., June.)

For psychasthenics, isolation is bad. They require work and re-education. (See Levy, and Review to appear in Jour. Abnor. Psychol., 1910). *(Neurasthenie et Neuroses).*

We must remember that every person has his normal ratio of endurance to fatigue. One man can march ten hours, another man only five. If the latter is made to march ten, a night's rest may not cure his fatigue. He is, for the time being, neurasthenic. The continuance of this, and especially when accompanied by worry, breaks down the metabolism, and we have the metabolic disorder which can be called a neurasthenia. There is no psychic element in this, of a direct kind, and we cannot too strongly protest against the view which confounds the psychogenetic disorder, hysteria, with the bodily fatiguability, to which we should limit the term neurasthenia. (Consult Williams' *Nature of Hysteria,* Int. Clin. 1908, N. O. Med. Jour., 1909, etc.)

Remember that a hysterical symptom is *nothing more than* "one induced by suggestion," (Babueske) and that it can usually be easily removed by suggestion also, or by persuasion, and kept away by a proper re-education, a psychotherapy of ideas. Suggested fatigue can be removed by suggestion, and is not true neurasthenia, in the treatment of which psychotperapy has part only in psychic complications. These might be compared to the postpneumonic empyema, which we have to treat by perforating the wall of the thorax. It would not be accurate to call paracentesis the treatment of pneumonia; and it is no more reasonable to invoke psychotherapy as the treatment of neurasthenia, a purely medical disease, which, however, often requires the neurologist for its diagnosis, and sometimes for the treatment of its hysterical complications. He does not, however, cure the neurast henia in removing its frequent psychic accompaniments, any more than does the surgeon who operates for an intestinal perforation of empyema remove the typhoid fever in which it originated.

Thus it is from clarity of diagnosis that precision of treatment arises.

